# COVID-19 Pandemic and Dental Practice

**DOI:** 10.1155/2020/8894794

**Published:** 2020-07-09

**Authors:** Anuraj Singh Kochhar, Ritasha Bhasin, Gulsheen Kaur Kochhar, Himanshu Dadlani

**Affiliations:** ^1^Former Orthodontist Max Hospital, Gurgaon, India; ^2^International Dentist Advanced Placement Program, Faculty of Dentistry, University of Toronto, Toronto, Canada; ^3^Pediatric & Preventive Dentistry, National Dental College & Hospital, Dera Bassi, Punjab, India; ^4^Periodontist, Max Hospital, Gurgaon, India

## Abstract

SARS-CoV-2, a virus causing severe acute respiratory syndrome, has inundated the whole world, generating global health concerns. There is a wildfire-like effect, despite the extensive range of efforts exercised by the affected countries to restrain the expanse of this pandemic, owing to its community spread pattern. Dental specialists in the upcoming days will likely come across patients with presumed or confirmed COVID-19 and will have to ensure stringent infection prevention and control to prevent its nosocomial spread. This paper strives to provide a brief overview of the etiology, incubation, symptoms, and transmission paradigms of this novel infection and how to minimize the spread in a dental healthcare setting. This review presents evidence-based patient management practice and protocols from the available literature to help formulate a contingency plan with recommendations, for the dental practices prior to patients' visit, during in-office dental treatment, and post-treatment, during the pandemic and after.

## 1. Introduction

A public health emergency intimidated the world in 2019, soon with a pandemic announced by the WHO with the emergence of an abstruse virus [1], the severe acute respiratory syndrome coronavirus (SARS-CoV-2). COVID-19, strangulating the world by spreading its tentacles in all spheres of life, initiated as a pneumonia outbreak in Wuhan, China. Coronaviruses belong to the Coronaviridae family, and SARS-CoV-2 based on the viral genome is a part of beta-coronavirus [2]. Coronaviruses are spherical in structure and have spiked glycoprotein on their surface that makes them appear like a crown, the reason for their name corona. Various studies have reported that human coronaviruses can remain viable on various inanimate surfaces from 2 hours to up to 9 days [3].

As of June 5, 2020, according to the World Health Organization (WHO), 2019-nCoV has involved 216 countries, over 6 million confirmed cases, and 387,155 confirmed deaths [4]. The aim of the publication is to provide a brief overview of the etiology, incubation, symptoms, and transmission paradigms of this novel infection and how to minimize the nosocomial spread in the dental healthcare setting. This review presents evidence-based patient management practice and protocols from the available literature to help formulate a contingency plan with recommendations, for the dental practices prior to patients' visit, during in-office dental treatment, and post-treatment, during the pandemic and after.

## 2. Materials and Methods

Relevant and swiftly evolving information regarding the SARS-CoV-2 and COVID-19 pandemic and any dental insinuations was obtained from electronic databases such as PubMed, PubMed Central, Medline, Scopus, and Google Scholar using the following search terms: “Coronavirus,” or “COVID-19,” or “SARS-CoV-2,” or “2019-nCoV,” separately combined with “incubation,” “transmission,” “symptoms,” “oral symptoms,” “dentistry,” “infection control,” “treatment,” “teledentistry,” and “protocol.” Peer-reviewed publications were given priority. Latest reports and guidelines from major health bodies such as the Centers for Disease Control and Prevention (CDC), World Health Organization (WHO), and major national dental associations and health regulatory bodies were also referred.

## 3. Results

Most recent peer-reviewed studies were given priority. However, most studies available were descriptive, small investigational studies, and narrative reviews due to the rapidly evolving information about the disease. A narrative synthesis was undertaken to provide a summary of the important aspects relevant to dental practitioners during and after the COVID-19 pandemic.

## 4. Discussion

### 4.1. Mode of Transmission

Initially, COVID-19 started as a zoonotic infection, followed by human-to-human transmission. SARS-CoV-2 uses angiotensin-converting enzyme-2 (ACE-2) which is found in the lower respiratory tract as its entry receptor. It is transmitted through both Flügge microdroplets due to direct proximity (a distance less than 2 metres and an exposure duration greater than 15 minutes) and core droplets that remain suspended in aerosol through coughing or sneezing by an infected person and possible transmission through fomites [3, 5].

Transmission of SARS-CoV-2 has been mainly described through inhalation/ingestion/direct mucous contact with saliva droplets [6–8] with the incubation period ranging from 5 to 14 days. The *R*_0_ (the basic reproductive number/infectious agent's epidemic potential) for SARS-CoV-2 ranges between 1.4 and 6.5, with an average of 3.28 [9, 10]. The disease may be highly infective, owing to viral shedding in early disease, SARS-CoV-2 transmission from asymptomatic carriers [11–13], and transmission through individuals during the incubation period [14].

### 4.2. Symptoms and Signs

COVID-19 may manifest as flu-like symptoms, ranging from dry cough, fever, sore throat, headache, lethargy, and diarrhea in few to troubled breathing, persistent pain or pressure in the chest, and bluish lips or face, which are emergency warning signs and necessitate immediate attention [6, 15, 16]. Dentists must be cognizant of oral findings and features, such as dysgeusia/ageusia, xerostomia, and exanthematous lesions like ulcers or blisters which might be early symptoms of COVID-19 and present before other typical clinical symptoms. Self-divulged loss of taste and smell is considered a much more substantial predictor of a positive COVID-19 diagnosis than self-diagnosed fever [17].

### 4.3. Diagnosis

A confirmed case is one which is positive for the 2019-nCoV by the real-time PCR test [18]. A single nasopharyngeal swab early in the course of disease is only 70% sensitive [19]. Hence, a single nasopharyngeal swab cannot be trusted blindly [17]. To et al. found the presence of the novel coronavirus in self-collected saliva specimens of 91.7% patients, which might be a viable source for diagnosis [20]. Rapid testing and monitoring of COVID-19 has been developed and approved by FDA, which will ease the pressure on hospitals, prevent the spread of infections, and save time [21].

### 4.4. Management in the Dental Office

Sound knowledge of the spread of SARS-CoV-2 is required to prevent its transmission in the dental practice. Aerosols are a predominant route for transmission of pathogens including SARS-CoV-2; therefore, stringent infection control measures are imperative [22–24]. Thus, specific recommendations for dental practice are required for patient screening and infection control strategies. Dentists must abide by the most recent recommendations from international, centralised/federal, and local public health authorities.

### 4.5. Pre-Visit Preparation

- Interdigitation of teledentistry which includes telescreening, teletriage, and teleconsulting is highly encouraged with telephone screening aimed to be the first point of contact between the patient and the dentist. Detailed medical history regarding the symptoms of COVID-19 (fever, cough and/or shortness of breath, sore throat, runny nose, diarrhea, lethargy discolouration of fingers or toes, rash of skin, and loss of taste and smell) must be investigated, and in case of any positive replies, in-office dental care should be delayed for 3 weeks except in case of dental emergencies [25, 26]. Teleconsultation via a live video is helpful in sharing health information such as radiographs and photographs through a secure electronic communications system with the practitioner to evaluate the patient's condition or provide virtual health service following an informed consent for the same [27].
- If needed, patients can be prescribed analgesics or topical agents via a teledentistry appointment itself.
- In lieu of the fomite spread, every surface in the reception area must be presumed to be a potential risk. All nonessential items such as dental display models, brochures, and magazines should be removed, and chairs in the waiting area should be placed 6 feet apart [28].
- All staff should change into different office clothing once they reach the office. Dentists, staff, and patients should be asked to hold-off on accessories such as bracelets, necklaces, and watches.
- Cleaning and disinfection and sterilization of the reception, waiting area, and equipment must be ensured [28].

### 4.6. Dental Office Visit Preparation

- Patients should be called/texted about their appointment and informed about the details of screening and in-office protocol [28]. Appointments should be staggered, and accompanying persons should not be encouraged, except for children or people with special needs. Before allowing any patient into the dental office, his/her temperature must be recorded. Hand sanitizer and masks should be made available for patients and attendants.
- Operatory preparation: a negative pressure/airborne infection isolation room should be allocated for treatment of any suspected COVID-19 patients to minimize the exposure of patients and staff [25]. The additional application of a portable high-efficiency particulate air (HEPA) filter may be considered [29], which is commonly present in air-purifiers and, hence, is readily available. Viability of SARS-CoV-2 on plastic and stainless steel was greater vis-a-vis copper and cardboard [30]. Hence, further research is recommended to substantiate the use of cardboard as barriers and copper instruments. Disposable devices could be considered; however, their disposal should be carried out carefully as infected medical waste [29].
- Hand hygiene: 80% ethanol or 75% 2-propanol as an Alcohol-Based Hand Rub (ABHR), against SARS-CoV and MERS-CoV, were found to be efficient. Hence, in dental practice, their use should be highly encouraged, and hands must be washed whenever visibly soiled [31].
- Personal Protective Equipment (PPE): Ti et al. advised the use of fit-tested N95 masks, gloves, overgown, and face/eye protection during aerosol-generating procedures on confirmed or suspected COVID-19 patients [5, 32]. The use of the double gloving technique with long sleeved gloves [29, 33], eyewear, including side shields or full-face-shields, and hair covers/hoods is highly recommended. It is suggested that donning a surgical mask or N95 is actually based on the size and dispersion of the respiratory secretions and the size of droplets known to be infectious for a specific contagion, rather than the actual size of the particle itself. Quan and colleagues researched salt-recrystallization-based virus deactivation systems. Their study shows that this filtration system provides high filtration efficiency and successfully deactivates multiple subtypes of adsorbed viruses [34].
- Pre-procedural mouthrinse with oxidative agents such as 1% hydrogen peroxide or 1% povidone iodine is considered to minimize the viral load [22, 28], thus helping in the reduction of aerosols and salivary pathogens concerned with 2019-nCoV. Other mouthrinses with chlorhexidine, Citrox, cyclodextrins combined with Citrox, and amphiphilic-cyclodextrin may prove to be beneficial but require further studies [35, 36].
- Xu et al. have shown that, in some cases of asymptomatic COVID-19 carriers, the virus may be residing in the salivary gland; hence, all patients should be considered carriers, and aerosol production must be reduced for all patients [37]. The use of 3-way syringes, high-speed handpieces, and ultrasonic scalers must be avoided as far as possible [3, 29]. If indispensable, antiretraction or electric friction grip handpieces must be used to prevent debris and fluids getting expelled or aspirated [1, 22]. Low- or high-volume suction can minimize aerosol production considerably [38, 39]. If restorative treatment is required, chemomechanical caries removal such as Carisolv [22] and papain gel [40] can be used. For restorations, silver diamine fluoride, biological restoration, and GIC can be utilized [41]. Wang et al. suggested that, in cases of acute pulpitis, periapical periodontitis, dental and orofacial trauma and infections, or any other dental emergency, patients and guardians should visit dental clinics with appropriate personal protection [42]. Rubber-dam use can be considered for aerosol-producing procedures, as airborne particles are reduced by 70% [38]. When required, the use of resorbable sutures over nonresorbable is suggested to minimize visits [37]. In radiography, extraoral radiography is favoured over intraoral techniques to reduce saliva production and gag reflex [1, 3, 22].

### 4.7. Post-Treatment

- Doffing of PPE: an appropriate doffing sequence and disposal in designated bags should be followed as per local biomedical waste protocols.
- Glasses and face-shields must be washed and disinfected after each procedure.
- ABHR must be used after each patient.
- Follow-up: all patients must be followed up after 7 days for any flu-like symptoms.
- Employee care: daily log for employees' temperature and symptoms must be made and reviewed periodically. An in/out daily log book needs to be maintained as to who all entered and left the office along with the date and time.
- Post-procedure disinfection and decontamination: all sterilizable instruments should be cleaned, disinfected, and sterilized expediently, while all disposables, whether used or not, should be presumed to be infected and discarded appropriately [29]. Concentrations between 62% and 71% of ethanol, 0.1 and 0.5% sodium hypochlorite, and 2% glutaraldehyde decreased coronavirus infectivity. An analogous effect is expected against the SARS-CoV-2 [42]. Hydrogen peroxide vaporizer can be utilized for operatory decontamination [32].
- Patients previously suffering from COVID-19 who have completed home isolation clearance can receive emergency dental care after fulfilling the latest CDC guidelines [43].

## 5. Conclusions

- In this unprecedented time, events are unfolding rapidly, and hence, all dental practitioners should be abreast with the latest news and guidelines in accordance with the regulatory bodies and IPAC protocol
- Asking patients about symptoms during reminder calls and rescheduling nonurgent appointments should be incorporated till the situation stabilizes
- The dental operatory should be well prepared, and stringent infection control and waste management protocols should be followed to reduce nosocomial infection
- Although currently catastrophic, even after the critical peak of the outbreak has been contained, management is needed as cases might still exist in the community for months and, perhaps, years
